# The translation and validation of the procrastination and precrastination traits scale in the modern Arabic Language

**DOI:** 10.1038/s41598-025-20963-1

**Published:** 2025-10-22

**Authors:** Haitham Jahrami, Waqar Husain, Hadeel Ghazzawi, Zahra Saif, Achraf Ammar, Khaled Trabelsi

**Affiliations:** 1 Department of Psychiatry, College of Medicine and Health Sciences, Arabian Gulf University, Manama, Bahrain; 2 Department of Humanities, COMSATS University Islamabad, Islamabad Campus, Park Road, Islamabad, Pakistan; 3 Nutrition and Food Technology Department, Agriculture School, The University of Jordan, Amman, Jordan; 4 Government Hospitals, Manama, Bahrain; 5 Department of Training and Movement Science, Institute of Sport Science, Johannes Gutenberg-University Mainz, 55099 Mainz, Germany; 6 Research Laboratory, Molecular Bases of Human Pathology, Faculty of Medicine of Sfax, University of Sfax, Sfax, LR19ES13, 3000 Tunisia; 7 Sport et Santé High Institute of Sport and Physical Education of Sfax Research Laboratory Education , University of Sfax , Motricité, EM2S, LR19JS01 Tunisia; 8 Department of Movement Sciences and Sports Training School of Sport Science , The University of Jordan , Amman, Jordan

**Keywords:** Procrastination, Precrastination, Arabic translation, Factor analysis, Reliability, Validity, Cross-cultural analysis, Health care, Psychology, Psychology

## Abstract

Procrastination and precrastination are distinct self-regulatory behaviors that influence individual functioning and well-being. Despite growing interest in these constructs, culturally adapted and psychometrically validated tools in Arabic-speaking contexts remain limited. This study addresses this gap by translating, validating, and evaluating the psychometric properties of the Arabic version of the Procrastination and Precrastination Traits Scale (PPTS). The present study aimed to examine the factorial structure, reliability, and convergent and divergent validity of the Arabic PPTS among Arabic-speaking adults and explore its associations with life satisfaction and standardized measures of procrastination. A sample of 1,000 participants (mean age = 27.93 years, standard deviation (SD) = 5.73; age range = 18–38) completed an online survey comprising the 18-item PPTS, the 12-item Pure Procrastination Scale (PPS), and the 5-item Satisfaction with Life Scale (SWLS). Descriptive analyses, confirmatory factor analysis (CFA), reliability testing, and bivariate correlations were conducted to assess psychometric performance. CFA supported a two-factor model with good fit indices (CFI = 0.939; TLI = 0.930; RMSEA = 0.05; SRMR = 0.04). All the items loaded significantly onto their respective factors: procrastination (items 1–10) and precrastination (items 11–18). Internal consistency was good for both subscales (procrastination: α = 0.861, ω = 0.861; precrastination: α = 0.788, ω = 0.803). Procrastination was positively correlated with PPS (r = 0.781, p < 0.001) and negatively correlated with SWLS (r = -0.475, p < 0.001). Conversely, precrastination was negatively associated with PPS (r = -0.405, p < 0.001) and positively associated with SWLS (r = 0.249, p < 0.001), supporting both convergent and divergent validity. The Arabic PPTS has adequate factorial validity, good reliability, and meaningful construct validity. It provides a culturally appropriate instrument for assessing behavioral tendencies of delay and hastiness, with implications for psychological assessment, educational interventions, and cross-cultural research on self-regulation and well-being.

## Introduction

Procrastination and precrastination are two distinct yet interconnected behaviors related to how individuals approach tasks and manage their time. Procrastination is defined as the voluntary delay of intended actions despite expecting negative consequences for the delay^1,2^. It can manifest in various forms, including delaying urgent tasks, postponing unpleasant chores, or waiting until the last minute to prepare for tests or deadlines^3,4^. Conversely, precrastination describes the tendency to complete tasks as soon as possible, even if it requires extra effort or is at the expense of efficiency^5–9^. This involves promptly tackling obligations and aiming to get things done ahead of schedule.

Both behaviors, when extreme, are considered potentially detrimental to mental health. Procrastination is associated with negative outcomes such as stress, health issues, academic underachievement, and lower well-being^10,11^. The newly developed Procrastination and Precrastination Traits Scale (PPTS) is a significant advancement^12^, as it is the first comprehensive psychometric tool designed to measure both of these traits simultaneously, addressing a notable gap in existing research that typically assesses them in isolation. The PPTS was developed using data from diverse continents, suggesting its potential for universal applicability.

Procrastination is a prevalent phenomenon worldwide, and its negative impacts extend to various aspects of life, including academic performance, work, and personal well-being^13,14^. In Saudi Arabia^15^, for example, a recent cross-sectional study revealed a high prevalence of chronic procrastination among adults, particularly students, with approximately 70% of participants reporting frequent procrastination. Common reasons cited for procrastination in this region include poor time management, boredom, distraction, and lack of motivation. These delays significantly impact personal life (55.1% reported a large or extreme impact) and work/studies (70% reported a large or extreme impact). Excessive screen time, often a procrastinating activity such as using social media or watching TV, is also highly prevalent, with more than half of the participants in one study spending more than 4 h daily on such activities, leading to feelings of guilt. Increased smartphone screen time has been linked to health issues such as insomnia, anxiety, depression, and higher BMI according to several recent meta-analyses^15^.

To provide a theoretical foundation for understanding procrastination and precrastination, this study is grounded in the Temporal Motivation Theory (TMT)^16^, which posits that task engagement is influenced by the interplay of expectancy, value, impulsiveness, and temporal proximity to rewards. Procrastination reflects a self-regulatory failure where individuals prioritize short-term rewards due to temporal discounting and low task expectancy, often leading to delayed action despite negative consequences^17^. In contrast, precrastination, as described by Rosenbaum et al. (2014), involves hastening task completion to reduce cognitive load, even at the cost of efficiency, reflecting a preference for immediate action^18^. By applying TMT, this study frames the Arabic PPTS as a tool to assess these contrasting self-regulatory behaviors, offering insights into their motivational and cognitive underpinnings within Arabic-speaking populations.

Given that Arabic is one of the most prevalent languages worldwide, spoken by over 450 million people^19^, there is a crucial need for culturally tailored and validated assessment tools. Understanding the significant impact of cultural and linguistic elements on the perception and treatment of psychological constructs, including addiction, necessitates the availability of such instruments. While other measures, such as the Arabic version of the Irrational Procrastination Scale^20,21^, have been translated and validated for Arabic-speaking populations to assess addictive behaviors, a comprehensive tool that simultaneously assesses both procrastination and precrastination has not been evaluated in Arabic. Therefore, translating and validating the PPTS into Arabic holds significant importance. This research would enable researchers, mental health providers, and educators to first gain a deeper understanding of how procrastination and precrastination manifest and interact within Arab societies; second, accurately assess these temporal self-regulation strategies in Arabic-speaking individuals; third, investigate the psychological and social consequences of these behaviors in the region; and finally, facilitate the development and implementation of evidence-based prevention and intervention strategies tailored to Arab settings.

## Methods

### Study design and setting

The study employed a cross-sectional correlational design, a widely accepted methodology for simultaneously assessing the prevalence of behavioral traits and evaluating the psychometric properties of instruments at a single point in time. The research was conducted among the general adult population residing within four Arabic-speaking countries, specifically Bahrain, Saudi Arabia, Jordan, and Tunisia (June 2025), to ensure direct cultural relevance and enhance the generalizability of the findings to the target linguistic group.

## Participants

The participants were recruited through a convenience sampling method, with the online questionnaire being widely distributed via various social media channels. Convenience sampling was chosen to efficiently reach a diverse group of Arabic-speaking adults across Bahrain, Saudi Arabia, Jordan, and Tunisia, aligning with established practices in cross-cultural psychometric research^22^. While this approach may not fully capture the diversity of Arabic-speaking populations, the sample size (*N* = 1,000) and inclusion of participants from four countries enhance the robustness and cultural relevance of the findings. To mitigate potential bias, we ensured broad dissemination of the survey link across various social media platforms to maximize demographic diversity. The inclusion criterion was Arabic-speaking adults aged 18 years or older who were also residents of Bahrain, Saudi Arabia, Jordan, or Tunisia at the time of the survey. This age demographic was particularly pertinent, as young adulthood is recognized as a crucial developmental period marked by significant life transitions and the emergence of complex self-regulatory behaviors related to time management. This age demographic spans emerging adulthood through early adulthood (18–38 years), representing a crucial developmental period marked by significant life transitions and the establishment of self-regulatory behaviors related to time management. The study sample consisted of 1,000 Arabic-speaking adults (mean age = 27.93 years, SD = 5.73; 61% female) recruited from Bahrain, Saudi Arabia, Jordan, and Tunisia using convenience sampling via social media platforms. In addition to age and gender, demographic data on educational level, employment status, and marital status were collected to provide a comprehensive sample profile. Results showed that 68% of participants held a bachelor’s degree or higher, 42% were employed, and 43% were married or engaged. The sample was distributed across the four countries as follows: Bahrain (*n* = 250, 25%), Saudi Arabia (*n* = 300, 30%), Jordan (*n* = 250, 25%), and Tunisia (*n* = 200, 20%). This sample size was considered robust for conducting the necessary psychometric analyses, adhering to recommendations for factor analyses^23^.

## Procedures

Data collection was managed through a secure online survey platform, which inherently ensured participant anonymity and voluntary participation. The survey package comprised the newly Arabic PPTS, a section for gathering demographic information, and other established scales deemed necessary for assessing convergent and divergent validity. Before the participants could commence the survey, an electronic informed consent form was explicitly presented to all prospective participants. This form clearly outlined the study’s objectives and the nature of participation and unequivocally stated their right to withdraw from the study at any point without incurring any consequences. The questionnaire link was broadly disseminated across popular social media platforms, replicating the successful data collection strategies employed in prior regional studies.

## Adaptation and translation processes

The translation and cultural adaptation of the PPTS into Arabic adhered to the internationally recognized “forward-backward-forward technique”, a gold standard for cross-cultural scale validation^24,25^. This methodical, multistep process was critical in ensuring both the linguistic accuracy and conceptual equivalence of the scale within the Arabic cultural context, aligning with the principles of good practice for translation and cultural adaptation^24,25^. The translation process incorporated an informal pilot phase. Specifically, the draft Arabic version of the scale was administered to a small group of Arabic-speaking adults (*n* = 30) drawn from all target countries to capture regional variation in dialect and cultural nuance. Participants represented both genders and a broad age range (18–45 years), and after completing the draft version, they provided qualitative feedback regarding item clarity, cultural appropriateness, and overall comprehensibility through structured debriefing questions. The results of the pilot indicated that all items were clearly understood, culturally appropriate, and linguistically accessible across different Arabic dialects. No substantive modifications were required to the translation, which provided reassurance regarding the instrument’s conceptual equivalence. Although this process was informal and therefore not designated as “cognitive interviewing,” it nonetheless served as an important quality check before full-scale administration.

The translation procedure encompassed the following distinct stages:

1. Preparation: Initially, the research team prepared the questionnaire. This involved carefully selecting and integrating the appropriate scales to ensure that the study’s aims were effectively addressed.
2. Forward translation and reconciliation: The original English version of the PPTS underwent independent translation into Arabic. This crucial step was performed by a specialized professional translator and two subject matter experts. These individuals possessed a profound understanding of both English and Arabic, enabling them to accurately capture the linguistic nuances and subtle meanings of the questionnaire’s items. A subsequent reconciliation process was then conducted to synthesize these independent translations into a single, cohesive Arabic version.
3. Back translation: Following forward translation, the reconciled Arabic version was translated back into English. This task was performed by a different professional translator who was entirely blinded to the original English version of the scale. This back-translation served as a critical quality control step, verifying that the translated content maintained fidelity to the original in terms of meaning, tone, and wording.
4. Harmonization: A collaborative harmonization phase was undertaken by the designated experts and the research team. During this stage, the back-translated English version was systematically compared against the original English questionnaire. Any identified discrepancies, inconsistencies, or areas necessitating cultural adjustment were discussed and resolved, and necessary modifications were implemented to achieve robust theoretical equivalence.
5. Proofreading and finalization: A conclusive Arabic version of the questionnaire was established through a rigorous proofreading process. This final review, involving both the researchers and experts, scrutinized all aspects of the scale, including grammar, syntax, clarity, and overall coherence, thereby guaranteeing the highest quality of the translated instrument.

## Measures

Procrastination and Precrastination Traits Scale (PPTS).

The central instrument utilized for this study was the PPTS^12^, which underwent translation and validation. The PPTS, developed by Husain et al. (2025), is an 18-item instrument designed to measure procrastination (Items 1–10) and precrastination (Items 11–18) tendencies using a 5-point Likert scale (1 = Never, 5 = Always). This innovative 18-item psychometric tool is noteworthy because it represents the first comprehensive instrument designed to simultaneously measure both procrastination and precrastination tendencies, addressing a significant gap in existing psychological research. The PPTS was structured into two distinct subscales: 10 items were dedicated to assessing various procrastination behaviors (e.g., “I delay getting on important tasks, even when I know I should not” and “I wait until the last minute to prepare for tests or deadlines”), whereas the remaining 8 items evaluated precrastination tendencies (e.g., “I complete tasks as soon as possible to get them out of the way” and “I keep my work and living spaces consistently neatly and organized”). The original PPTS^12^, validated across diverse samples from six continents, exhibited a robust two-factor structure with strong model fit (CFI = 0.951, TLI = 0.943, RMSEA = 0.047). Internal consistency was high, with Cronbach’s alpha of 0.873 for procrastination and 0.801 for precrastination, and McDonald’s omega values of 0.875 and 0.804, respectively. Convergent validity was supported by correlations with the Pure Procrastination Scale (*r* = 0.792 for procrastination, *r* = −0.389 for precrastination), while divergent validity was confirmed through associations with life satisfaction measures.

Pure Procrastination Scale (PPS).

This scale measures procrastination behaviors across various life domains^26^. It comprises 12 items rated on a 5-point Likert scale ranging from “very rarely or does not represent me” to “very often or always represents me”. The items cover aspects such as delaying the start or completion of tasks, postponing decisions, and failing to meet deadlines. The PPS has demonstrated good psychometric properties, with Cronbach’s alpha values typically exceeding 0.75 in various studies.

Satisfaction with Life Scale (SWLS).

This is a well-known and widely utilized measure for assessing overall cognitive evaluations of life satisfaction^27^. It consists of 5 items, with responses on a 7-item Likert scale ranging from “strongly disagree” to “strongly agree”. Higher scores on the SWLS indicate higher levels of life satisfaction, and its reliability and validity have been demonstrated in numerous studies globally.

### Statistical analysis

All the statistical analyses were performed via specialized software packages, namely, STATA 17 and R, for statistical computing 4.5.1. Prior to any inferential analyses, data integrity checks were conducted. This involved rigorous screening procedures to identify and manage missing values, detect outliers (e.g., using extreme z scores), and flag potential automated or careless responses, such as repetitive answer sequences, monotonous patterns, or implausibly rapid survey completion times (defined as less than 2 s per item). Assumptions for statistical tests, including normality (e.g., via skewness and kurtosis indices) and multicollinearity (e.g., using variance inflation factors), were assessed. The psychometric evaluation involved several key statistical procedures.

Confirmatory Factor Analysis (CFA): To assess the underlying factor structure of the Arabic PPTS definitively, a CFA was performed. This analysis systematically compared the fit of a hypothesized two-factor model (representing procrastination and precrastination as distinct but related constructs) against a simpler one-factor model. Model fit was evaluated via conventional indices, including the normed model chi-square (χ²/df), the Root Mean Square Error of Approximation (RMSEA), the Tucker‒Lewis Index (TLI), and the Comparative Fit Index (CFI). Target values for good model fit (e.g., χ²/df ≤ 5, RMSEA ≤ 0.08, and CFI/TLI ≥ 0.95) guided interpretation. Additionally, the average variance extracted (AVE) was computed to provide further evidence for convergent validity at the construct level (values of 0.5 or more were considered adequate).

Reliability Analysis: The internal consistency of the Arabic PPTS was assessed for both its procrastination and precrastination factors. This was determined by calculating the coefficient omega (ω) and Cronbach’s alpha (α). Acceptable reliability was indicated by values exceeding 0.70, with values above 0.80 considered good.

Measurement invariance: To ensure that the scale functioned equivalently across different demographic groups, multigroup CFA was conducted to examine the sex invariance of the PPTS scores. This involved assessing configural, metric, and scalar levels of invariance, confirming that the scale’s structure and item meanings were consistent between male and female Arabic-speaking participants. Invariance was supported if the ΔCFI was ≤ 0.010, the ΔRMSEA was ≤ 0.015, or the ΔSRMR was ≤ 0.010.

Convergent and divergent validity: The scores from the Arabic PPTS were correlated with scores from the established measures of life distress (SWLS) scale and known procrastination scale (PPS). The intercorrelations between procrastination and precrastination factors themselves were also scrutinized to confirm their distinct yet interrelated nature.

Multi-group CFA: To evaluate the cross-cultural validity of the Arabic PPTS across the four participating countries, we conducted multi-group CFA to test measurement invariance across both sex and country. Invariance testing followed the sequential procedure of evaluating configural, metric, and scalar invariance. Model fit was assessed using established cut-off values for CFI, RMSEA, and SRMR. Changes in model fit indices (ΔCFI ≤ 0.010, ΔRMSEA ≤ 0.015, ΔSRMR ≤ 0.010) were used to determine whether increasingly restrictive models retained equivalence across groups.

Decision Rules: To ensure proper of the Arabic PPTS’s psychometric properties, statistical analyses were guided by established decision rules. For assessing normality, skewness and kurtosis values between − 2 and + 2 were considered indicative of approximate normality^28^. For CFA, good model fit was defined by χ²/df ≤ 5, CFI and TLI ≥ 0.95, RMSEA ≤ 0.08, and SRMR ≤ 0.08^29^. Reliability was evaluated using Cronbach’s alpha and McDonald’s omega, with values ≥ 0.70 deemed acceptable and ≥ 0.80 considered good^30^. For measurement invariance across sex, educational status, marital status, and country, invariance was supported if ΔCFI ≤ 0.010, ΔRMSEA ≤ 0.015, and ΔSRMR ≤ 0.010^31^.

Additional Analysis: To comprehensively evaluate the Arabic PPTS’s item performance, additional analyses were conducted, including corrected item-total correlations and exploratory factor analyses (EFA) with forced single-factor solutions for each subscale. Corrected item-total correlations assessed item discrimination, with a threshold of *r* ≥ 0.30 indicating acceptable item contribution^28^. Single-factor EFAs were performed separately for the procrastination (Items 1–10) and precrastination (Items 11–18) subscales to examine item loadings and explained variance, with loadings ≥ 0.50 considered adequate^28^.

For all analyses, a p value of less than 0.05 was considered statistically significant.

## Ethical considerations

Prior to the commencement of any data collection, full ethical approval was sought and obtained from the Institutional Review Board (IRB) of the Psychiatric Hospital, Government Hospitals, Bahrain, mirroring the stringent ethical protocols observed in prior research within the region.

All research procedures were conducted in strict adherence to the ethical principles outlined in the Declaration of Helsinki and all pertinent local guidelines and regulations. Participation in this study was entirely voluntary. Informed consent was electronically secured from all eligible participants before they engaged with the survey, ensuring that they were fully aware of the study’s objectives and their absolute right to withdraw at any point without any consequences. The utmost confidentiality and anonymity of all participant data were rigorously maintained throughout the entire duration of the study.

To address potential concerns regarding the reliance on self-report measures, particularly for constructs such as procrastination and precrastination that may be subject to social desirability or inaccurate self-assessment, the study employed anonymous online surveys to minimize reporting biases. Anonymity has been shown to enhance response honesty and reduce social desirability effects in psychological research^22^. In addition, the Arabic PPTS was administered alongside established instruments, including the Pure Procrastination Scale and the Satisfaction with Life Scale, to provide evidence of convergent and divergent validity, thereby indirectly supporting the accuracy of self-reported responses. While behavioral or clinical validation was beyond the scope of the present study, this limitation is acknowledged, and future research should incorporate objective behavioral measures—such as task completion times, performance-based indicators, or observational data—to further strengthen the validity of the Arabic PPTS and reduce reliance on self-report data alone.

## Results

The present study assessed the psychometric properties of the Arabic version of the Procrastination and Precrastination Traits Scale (PPTS) in a sample of 1,000 participants. Descriptive statistics revealed that the mean age of the participants was 27.93 years (SD = 5.73), with a range from 18 to 38 years (Table 1).

**Table 1:** Descriptive statistics of the study variables (*N* = 1000).

Variable	Mean	SD	Minimum	Maximum	Skewness	Kurtosis
Age (Years)	27.93	5.73	18.00	38.00	0.03	−1.21
Procrastination	3.34	0.90	1.00	5.00	−0.31	−0.72
Precrastination	3.17	0.87	1.00	5.00	−0.11	−0.78
PPS	48.32	20.75	12.00	84.00	0.04	−1.23
SWLS	20.58	7.93	7.00	35.00	0.00	−1.19

**Notes: **Procrastination and precrastination are the two subscales of the Procrastination and Precrastination Traits Scale (18 items). PPS = the 12-item Pure Procrastination Scale. SWLS = Satisfaction with Life Scale.

The distributions for all the variables were approximately normal, with skewness and kurtosis values ranging between − 0.31 and 0.04 for skewness and between − 1.23 and − 0.72 for kurtosis (Table 1).

The mean scores for the psychological variables were as follows: procrastination (M = 3.34, SD = 0.90), precrastination (M = 3.17, SD = 0.87), pure procrastination scale (PPS; M = 48.32, SD = 20.75), and satisfaction with life scale (SWLS; M = 20.58, SD = 7.93). These findings suggest that participants, on average, exhibited moderate levels of procrastination and precrastination behaviors and reported midrange levels of life satisfaction (Table 1).

To evaluate the factorial validity of the 18-item Arabic version of the PPTS, confirmatory factor analysis (CFA) was conducted via maximum likelihood estimation (Table 2). The hypothesized two-factor model demonstrated acceptable to good overall fit to the data. Fit indices included: χ²(134) = 467.374, *p* < 0.001; normed chi-square χ²/df = 3.49; CFI = 0.939; TLI = 0.930; RMSEA = 0.05 (90% CI [0.045, 0.055], p-close = 0.507); and SRMR = 0.04. The normed chi-square was within acceptable range (≤ 5), RMSEA demonstrated good fit (< 0.06), while CFI and TLI showed acceptable fit (≥ 0.90), and SRMR was excellent (< 0.05). These indices collectively support the adequacy of the two-factor structure. All factor loadings were statistically significant (*p* < 0.001), with values ranging from 0.24 to 0.74. However, Item 15 demonstrated a problematic factor loading of 0.24, which falls well below the conventional threshold of 0.50 for acceptable factor loadings in CFA^32^. All other items showed acceptable to strong factor loadings (range: 0.50–0.74), with most items demonstrating loadings above 0.57.

**Table 2:** Confirmatory factor analysis and reliability analysis of the Arabic version of the procrastination and precrastination traits scale (18 items), *N* = 1000 participants.

Factor loadings						Residual variances			
Factor	Indicator	Value	SE	z value	*p* value	Value	SE	z value	*p* value
**Factor 1**	Item1	0.64	0.039	21.19	< 0.001	0.60	0.051	20.08	< 0.001
	Item2	0.50	0.043	15.75	< 0.001	0.75	0.066	21.33	< 0.001
	Item3	0.58	0.043	18.83	< 0.001	0.67	0.063	20.68	< 0.001
	Item4	0.57	0.043	18.54	< 0.001	0.68	0.064	20.79	< 0.001
	Item5	0.65	0.039	22.02	< 0.001	0.57	0.049	19.96	< 0.001
	Item6	0.69	0.037	23.58	< 0.001	0.52	0.044	19.38	< 0.001
	Item7	0.59	0.043	19.31	< 0.001	0.65	0.062	20.63	< 0.001
	Item8	0.74	0.037	25.95	< 0.001	0.45	0.040	18.39	< 0.001
	Item9	0.67	0.038	22.71	< 0.001	0.55	0.045	19.72	< 0.001
	Item10	0.59	0.042	19.39	< 0.001	0.65	0.060	20.68	< 0.001
**Factor 2**	Item11	0.68	0.043	22.55	< 0.001	0.54	0.059	18.43	< 0.001
	Item12	0.64	0.043	20.61	< 0.001	0.60	0.060	19.31	< 0.001
	Item13	0.68	0.039	22.37	< 0.001	0.54	0.050	18.56	< 0.001
	Item14	0.50	0.044	15.41	< 0.001	0.75	0.067	20.89	< 0.001
	Item15	0.24	0.048	6.99	< 0.001	0.94	0.084	22.08	< 0.001
	Item16	0.56	0.043	17.59	< 0.001	0.69	0.063	20.33	< 0.001
	Item17	0.65	0.040	21.15	< 0.001	0.58	0.051	19.06	< 0.001
	Item18	0.63	0.042	20.26	< 0.001	0.61	0.059	19.47	< 0.001

**Notes**: Method: Confirmatory factor analysis was conducted via maximum likelihood estimation. Factor Structure: Factor 1 = Procrastination (Proc = Items 1–10) Factor 2 = Precrastination (Prec = Items 11–18). Model Fit Statistics: Chi-square Test: χ² = 467.374, df = 134, *p* < 0.001 Normed Chi-square: χ²/df = 3.49 Comparative Fit Index (CFI): 0.939 Tucker-Lewis Index (TLI): 0.930 Root Mean Square Error of Approximation (RMSEA): 0.05 RMSEA 90% CI: [0.045, 0.055] RMSEA p-value: 0.507 Standardized Root Mean Square Residual (SRMR): 0.04.

Comprehensive item analysis revealed that Item 15 in the precrastination subscale performed poorly across multiple indicators: a corrected item-total correlation of 0.19 (below the 0.30 threshold), a single-factor EFA loading of 0.26 (below the 0.50 threshold), and weak inter-item correlations with other precrastination items (*r* = 0.12–0.24). In contrast, procrastination items (1–10) showed adequate item-total correlations (*r* = 0.52–0.68) and EFA loadings (0.56–0.75, explaining 42.3% variance), while precrastination items (11–14, 16–18) had item-total correlations of 0.45–0.62 and EFA loadings of 0.48–0.71 (explaining 35.8% variance). Removing Item 15 resulted in a 17-item scale with improved precrastination subscale properties: Cronbach’s α increased to 0.812 (from 0.788), McDonald’s ω to 0.821 (from 0.803), AVE to 0.398 (from 0.342), and mean inter-item correlation to 0.34 (from 0.28). A CFA for the 17-item model showed enhanced fit: χ²(118) = 389.45, *p* < 0.001, χ²/df = 3.30, CFI = 0.948, TLI = 0.940, RMSEA = 0.048, SRMR = 0.038.

Reliability analyses indicated good internal consistency for both subscales: Cronbach’s alpha and McDonald’s omega were α = 0.861 and ω = 0.861 for procrastination and α = 0.788 and ω = 0.803 for precrastination.

Corrected item-total correlations for the procrastination subscale (Items 1–10) ranged from 0.52 to 0.68, all exceeding the 0.30 threshold, indicating good item discrimination. For the precrastination subscale (Items 11–18), correlations ranged from 0.45 to 0.62 for Items 11–14 and 16–18, but Item 15 showed a weak correlation of 0.19, below the acceptable threshold. Single-factor EFA for the procrastination subscale yielded loadings of 0.56 to 0.75, explaining 42.3% of the variance, with all items loading adequately (> 0.50). For the precrastination subscale, EFA loadings ranged from 0.48 to 0.71 for Items 11–14 and 16–18, but Item 15 had a low loading of 0.26, consistent with CFA results, with the subscale explaining 35.8% of the variance.

Convergent and divergent validity (Table 3) were examined through bivariate correlations with established measures. Average Variance Extracted (AVE) was calculated to assess convergent validity at the construct level. The procrastination factor demonstrated an AVE of 0.386, while the precrastination factor showed an AVE of 0.342. Both values fell below the conventional threshold of 0.50 typically recommended for adequate convergent validity^33^, suggesting that the factors explain less than half of the variance in their respective indicators. The procrastination factor demonstrated a large positive correlation with the PPS (r = 0.781, p < 0.001, large effect size), supporting strong convergent validity. To evaluate discriminant divergent validity, Average Shared Variance (ASV) and Maximum Shared Variance (MSV) were calculated alongside AVE values^32^. Procrastination factor: AVE = 0.386, MSV = 0.610, ASV = 0.375 Precrastination factor: AVE = 0.342, MSV = 0.289, ASV = 0.172”. The procrastination factor also showed a large negative correlation with life satisfaction (r = −0.475, p <.001, large effect size), indicating good divergent validity. The precrastination factor showed a large negative correlation with the PPS (r = −0.405, p < 0.001, medium effect size) and a small but significant positive correlation with SWLS (r = 0.249, p < 0.001, small effect size). The moderate-to-large negative correlation between the two PPTS factors (r = −0.538, p < 0.001, large effect size) confirms they represent distinct but related constructs.

**Table 3:** Convergent and divergent validity of the Arabic version of the procrastination and precrastination traits scale (18-items) with the 12-item pure procrastination scale (PPS) and the satisfaction with life scale (SWLS) *N* = 1000 participants.

Variables	Factor 1	Factor 2	PPS	SWLS
**Factor 1 (Procrastination)**	---			
**Factor 2 (Precrastination)**	−0.538* (Large)	---		
**PPS**	0.781* (Large)	−0.405* (Medium)	---	
**SWLS**	−0.475* (Large)	0.249* (Small)	−0.398* (Medium)	---
**Cronbach’s Alpha (α)**	0.861	0.788	0.912	0.884
**McDonald’s Omega (ω)**	0.861	0.803	0.914	0.886
**Number of Items**	10	8	12	5

No significant differences in procrastination or precrastination were observed across genders. The correlations of both procrastination and precrastination with age were statistically insignificant.

The multi-group CFA supported measurement invariance of the Arabic PPTS across both sex and country. Configural invariance indicated that the underlying factor structure was consistent across all groups. Metric invariance was achieved, suggesting equivalence in factor loadings, and scalar invariance was established, supporting equivalence in item intercepts. Fit indices remained within acceptable thresholds across all models, with changes between increasingly constrained models falling below recommended cut-offs (ΔCFI ≤ 0.010, ΔRMSEA ≤ 0.015, ΔSRMR ≤ 0.010). These findings indicate that the Arabic PPTS demonstrates stable psychometric properties across the four countries despite potential cultural variation.

These descriptive, factorial, and correlational analyses provide evidence supporting the reliability and construct validity of the Arabic version of the Procrastination and Precrastination Traits Scale. The scale effectively captures the bidirectional tendencies of delay and premature action and demonstrates meaningful relationships with established measures of procrastination and life satisfaction.

## Discussion

The present study aimed to examine the psychometric properties of the Arabic version of the PPTS, with a particular focus on its factorial structure, reliability, and convergent/divergent validity. The findings provide good evidence that the 18-item scale effectively captures procrastination and procrastination among Arabic-speaking adults. This study contributes to the growing body of cross-cultural research on self-regulatory behaviors by offering a validated instrument for use in Arabic-speaking populations.

The confirmatory factor analysis supported the hypothesized two-factor structure of the PPTS, comprising 10 items measuring procrastination and 8 items assessing precrastination. All factor loadings were statistically significant, and most items demonstrated moderate to strong loadings, with the exception of Item 15, which had a weak loading (λ = 0.24). The overall model fit indices, such as CFI (0.939), TLI (0.930), and RMSEA (0.05), fell within acceptable to excellent ranges, suggesting that the scale’s factorial structure is adequate and replicable in Arabic contexts. Item 15 (“ I don’t postpone making decisions, even on small matters.”) on the precrastination subscale exhibited a notably low factor loading (λ = 0.24), item-total correlation (*r* = 0.19), and weak inter-item correlations (*r* = 0.12–0.24). While the low loading did not compromise the overall factor structure, it indicates that this item may contribute less to the measurement of the underlying construct. This suggests potential issues with the item’s cultural or linguistic clarity in the Arabic context. One potential explanation is that the behavioral tendency to “get things done ahead of schedule” may not be universally emphasized or framed in the same way across Arabic-speaking cultures, where social and contextual norms around task completion and time management might differ. Additionally, the phrasing may have been interpreted variably by participants, with some perceiving it as normative or habitual rather than reflective of individual differences in precrastination. Removing Item 15 improved the precrastination subscale’s reliability (α = 0.812, ω = 0.821), AVE (0.398), and model fit (CFI = 0.948, RMSEA = 0.048), supporting a 17-item Arabic PPTS for future use. These findings, consistent with the original English PPTS’s item refinement needs ^12^, indicate that revising or removing Item 15 could enhance the scale’s validity and applicability for Arabic-speaking populations^12,28^. To address this issue, we recommend that future studies employ qualitative approaches, such as cognitive interviewing or focus groups, to explore how participants interpret this item. Based on such findings, revisions could involve rewording the item to better align with culturally relevant examples of proactive task completion or considering replacement with a more contextually appropriate behavior. Future validation studies should prioritize revising Item 15 or developing alternative items that better capture precrastination tendencies. Factor loadings should consistently exceed 0.50, and items with loadings below 0.40 should be considered for removal or substantial revision.

The internal consistency estimates were strong. Cronbach’s alpha and McDonald’s omega exceeded the recommended threshold of 0.70 for both subscales. The slightly lower AVE values for the two factors (0.386 and 0.342) suggest that while the internal consistency is high, the shared variance among items may be somewhat limited. However, such AVE thresholds are not uncommon in research involving behavioral tendencies, where trait expressions may vary contextually^28^.

Convergent validity was well established through a strong positive correlation between the procrastination factor and the PPS (*r* = 0.781, *p* < 0.001. In contrast, the precrastination factor was negatively associated with PPS (*r* = −0.405), reaffirming its role as a theoretically distinct behavioral pattern characterized by hasty task initiation and premature action. Furthermore, the divergent validity of the PPTS was demonstrated through its correlation with life satisfaction. Procrastination was negatively related to satisfaction with life (*r* = −0.475), in line with prior evidence indicating that habitual delay undermines subjective well-being and goal achievement^34^. Conversely, precrastination was modestly but positively associated with life satisfaction (*r* = 0.249), suggesting that a proactive approach to task completion may serve adaptive functions under certain circumstances. The divergent validity analysis reveals some concerns beyond the poor convergent validity. The procrastination factor’s AVE (0.386) was exceeded by both its MSV (0.610) and ASV (0.375), indicating that this factor shares more variance with other constructs than with its own indicators. This is particularly problematic given the very high correlation with PPS (*r* = 0.781), suggesting potential construct overlap rather than distinct measurement.

The slightly lower AVE values for the two factors (procrastination = 0.386; precrastination = 0.342) reported in the Results section suggest that while the internal consistency is high, the convergent validity at the construct level requires improvement. These AVE values fall below the recommended threshold of 0.50^33^, indicating that the factors explain less than half of the variance in their indicators. This limitation may be partly attributable to the poor performance of Item 15, which showed weak factor loading and may be diluting the precrastination factor’s convergent validity.

To strengthen the evidence for construct distinctness, the study explicitly examined divergent validity and the potential overlap between procrastination and precrastination. Divergent validity was demonstrated through correlations with the SWLS: procrastination showed a moderate negative correlation, consistent with its association with reduced well-being, while precrastination exhibited a weak positive correlation, suggesting adaptive tendencies in some contexts. Furthermore, the moderate negative correlation between procrastination and precrastination supports their conceptualization as related but distinct constructs, aligning with prior evidence^6^. CFA further reinforced this distinction, with a two-factor model demonstrating good fit, and the HTMT indicating acceptable divergent validity. Collectively, these findings affirm that the Arabic PPTS can effectively differentiate procrastination and precrastination among Arabic-speaking populations. Nevertheless, future research should incorporate additional divergent validity indicators, such as impulsivity or conscientiousness, to further refine construct boundaries and enhance cross-cultural applicability.

The moderate negative correlation between the two factors (*r* = −0.538, *p* < 0.001) suggests they are related but not purely bipolar, with only 29% shared variance, aligning with the independence model proposed by Rosenbaum et al. (2019)^6^. This indicates that individuals may exhibit both traits across different contexts, such as procrastinating on unpleasant tasks while precrastinating on urgent or enjoyable ones. Compared to the original English PPTS (*r* = −0.43)^12^, our findings reinforce that separate subscales are necessary to capture distinct behavioral tendencies, as low procrastination scores do not automatically imply high precrastination. This dual measurement approach enhances the scale’s utility for identifying context-specific patterns and tailoring interventions, acknowledging that both extreme procrastination and precrastination can be maladaptive.

The establishment of measurement invariance across sex and country provides strong evidence that the Arabic PPTS is psychometrically consistent across diverse Arabic-speaking contexts. Despite cultural and linguistic variations between Bahrain, Saudi Arabia, Jordan, and Tunisia, the scale demonstrated configural, metric, and scalar invariance, confirming that participants across these countries interpret and respond to items in a comparable manner. This supports the generalizability of the Arabic PPTS for cross-national research and clinical applications in Arabic-speaking populations. Nonetheless, while invariance was supported in this study, future research with larger and more diverse samples from additional Arabic-speaking countries is warranted to further substantiate the instrument’s robustness across the broader Arab world.

The current study focused on adults in emerging and early adulthood (ages 18–38, M = 27.93), which represents a critical period for understanding self-regulatory behaviors as individuals transition through educational completion, career establishment, and personal relationship formation. The study revealed no statistically significant differences in procrastination or precrastination based on gender, nor were there any significant correlations between these traits and age. These findings contribute to an ongoing debate in the literature regarding the influence of demographic variables on self-regulatory behaviors. The lack of gender differences in procrastination contradicts some earlier studies suggesting that men tend to procrastinate more than women do^11,35^. However, other studies have failed to replicate these gender differences, especially in large and culturally diverse samples. For example, Svartdal and Steel (2017), using data from multiple countries, reported that gender differences in procrastination were either negligible or inconsistent across contexts^36^. In line with these findings, our results support the growing view that gender may not be an adequate predictor of procrastination when assessed across heterogeneous adult populations. The same applies to procrastination, which is a relatively novel construct showing limited and inconclusive evidence for gender effects in prior research^18^. The finding that age was not significantly correlated with either procrastination or precrastination in this sample also warrants attention. Although studies have shown that procrastination decreases with age, particularly in adolescence and early adulthood^37^, the current study focused on a relatively narrow age range (18–38 years, M = 27.93), which may explain the absence of significant effects. It is also essential to consider sociocultural factors that may moderate or mask demographic patterns in procrastination.

Overall, the validation of the PPTS in Arabic supports the growing recognition that procrastination and precrastination are not merely opposite ends of a single continuum but are independent behavioral tendencies with distinct psychological profiles and outcomes^6^.

### Implications

The validated Arabic version of the PPTS provides researchers and practitioners with a culturally and linguistically appropriate instrument for assessing procrastination and precrastination. It has potential applications in educational, clinical, and occupational settings where procrastination and precrastination may impact performance, well-being, or treatment adherence. Interventions aimed at reducing maladaptive procrastination or regulating excessive precrastination can be better tailored when informed by reliable, culturally sensitive assessments.

### Limitations

Our sample was concentrated in emerging and early adulthood (18–38 years), which may limit generalizability to middle-aged and older adults who may demonstrate different patterns of procrastination and precrastination as life responsibilities and priorities change. Future studies should examine the scale’s applicability across broader age ranges.

The cross-sectional design and reliance on convenience sampling introduce limitations that may restrict causal inferences and affect the external validity of the Arabic PPTS. While the large and inclusion of participants from four countries enhance the cultural relevance of the findings, the cross-sectional approach precludes conclusions regarding causal relationships between procrastination and precrastination traits. Additionally, convenience sampling via social media may not fully capture the diversity of the broader Arabic-speaking population, potentially introducing selection bias. Therefore, future research should employ longitudinal designs to examine causal pathways and utilize stratified or probability-based sampling methods to improve representativeness, thereby strengthening confidence in the psychometric robustness and cross-cultural applicability of the Arabic PPTS.

The sample’s relatively narrow age range represents a limitation that may restrict the generalizability of findings, particularly to older adults whose procrastination and precrastination behaviors may differ. While this age range was chosen to capture a developmental period where self-regulatory behaviors are especially salient, prior research suggests that such behaviors may decline or manifest differently across the lifespan^37^. Accordingly, the results should be interpreted with caution, and future research should include broader age groups to examine potential age-related variations in procrastination and procrastination.

The AVE values for the procrastination and precrastination subscales fall below the recommended threshold of 0.50, indicating a potentially limited convergent validity. While AVE provides an index of the proportion of variance captured by a latent construct relative to measurement error, values below 0.50 are not uncommon in behavioral research, particularly for multifaceted constructs such as procrastination and precrastination that are context-dependent and influenced by situational and cultural factors^28^. Future studies should consider refining item content and including additional behavioral or performance-based measures to strengthen shared construct variance.

The exclusive reliance on self-report measures introduces potential biases, including social desirability and inaccurate self-perception, which may affect the fidelity of findings. Although convergent and divergent validity with established scales provides indirect support, future research should incorporate objective behavioral or performance-based measures, such as task completion times or observational assessments, to corroborate self-reports and enhance the psychometric robustness of the Arabic PPTS.

Finally, although multi-group analyses indicated no gender differences, sociocultural factors specific to Bahrain, Saudi Arabia, Jordan, and Tunisia—such as gender roles, educational expectations, and societal pressures—may mask true demographic effects; examining these cultural moderators through qualitative or mixed-methods approaches could provide deeper insight into the expression of procrastination and precrastination across different sociocultural contexts.

## Conclusion

This study presents compelling evidence for the factorial validity, reliability, and construct validity of the Arabic version of the PPTS. The findings confirm that procrastination and precrastination are empirically distinguishable constructs with unique psychological correlates. By providing a validated measure suited for Arabic-speaking populations, this research enhances the cross-cultural applicability of self-regulation theories and offers valuable insights for future studies exploring adaptive and maladaptive forms of behavioral timing.

**Fig. 1:**
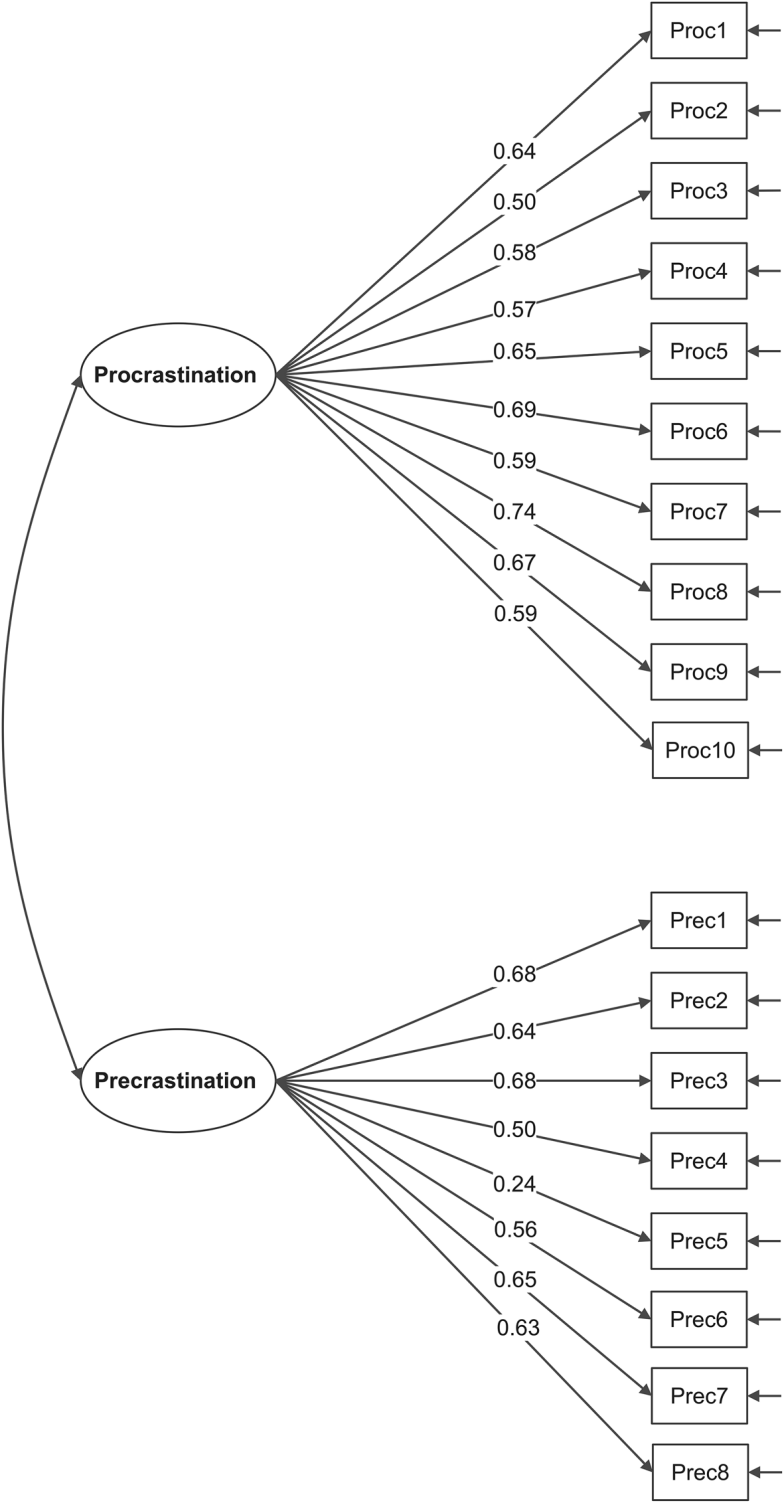
Path diagram of the Arabic version of the procrastination and precrastination traits scale. Legend for Fig. 1: Factor 1 = Procrastination (Proc = Items 1–10) Factor 2 = Precrastination (Prec = Items 11–18). Procrastination Items: (1) I delay getting started on important tasks, even when I know I shouldn’t. (2) I keep putting off unpleasant chores or obligations. (3) I find myself aimlessly browsing instead of working. (4) I wait until the last minute to prepare for tests or deadlines. (5) I tell myself I’ll get more done “tomorrow” rather than following through today. (6) I waste time on unimportant distractions instead of priorities. (7) I procrastinate on health habits like exercise, diet, or appointments. (8) I unnecessarily delay making even small decisions. (9) I leave emails, messages or calls unanswered for too long. (10) My spaces become cluttered and disorganized from postponing cleaning/organizing. Precrastination Items: (11) I complete tasks as soon as possible to get them out of the way. (12) I take care of chores or obligations immediately rather than delaying. (13) I tackle unpleasant tasks right away without postponing. (14) I try to get things done ahead of schedule whenever possible. (15) I don’t postpone making decisions, even on small matters. (16) I reply to messages, emails, or calls promptly without delay. (17) I keep my work and living spaces consistently neat and organized. (18) I don’t wait for deadlines to start on important tasks or goals.

## Data Availability

The corresponding author can provide the data upon request.

## Abbreviations

AIC: Akaike information criterion
CFI: Comparative fit index
CFA: Confirmatory factor analysis
EFA: Exploratory factor analysis
GFI: Goodness-of-fit index
KMO: Kaiser–Meyer–Olkin
MFI: McDonald fit index
NNFI: Bentler–bonett nonnormed fit index
PPTS: Procrastination and precrastination traits scale
PPS: Pure Procrastination Scale
RMSEA: Root mean square error of approximation
SE: Standard error
SD: Standard deviation
SRMR: Standardized root mean square residual
SWLS: Satisfaction with Life Scale
TLI: Tucker‒lewis index
